# Development of CIDEA reporter mouse model and its application for screening thermogenic drugs

**DOI:** 10.1038/s41598-021-97959-0

**Published:** 2021-09-16

**Authors:** Yeonho Son, Cheoljun Choi, Cheol Song, Hyeonyeong Im, Yoon Keun Cho, Ju Seung Son, Sungug Joo, Yoonjoe Joh, Young Jae Lee, Je Kyung Seong, Yun-Hee Lee

**Affiliations:** 1 College of Pharmacy and Research Institute of Pharmaceutical Sciences, Seoul National University, Seoul, Republic of Korea; 2 Korea Mouse Phenotyping Center (KMPC) and Lee Gil Ya Cancer and Diabetes Institute, Gachon University, Inchon, Republic of Korea; 3 Department of Biochemistry, College of Medicine, Gachon University, Inchon, Republic of Korea; 4 Korea Mouse Phenotyping Center (KMPC) and Laboratory of Developmental Biology and Genomics, Research Institute for Veterinary Science, and BK 21 PLUS Program for Creative Veterinary Science Research, College of Veterinary Medicine, Seoul National University, Seoul, Republic of Korea

**Keywords:** Drug discovery, Drug screening, Phenotypic screening, Obesity

## Abstract

Cell death-inducing DNA fragmentation factor-like effector A (CIDEA) is a lipid droplet-associated protein and is a known marker of the thermogenic capacity of brown/beige adipocytes. To monitor the expression of CIDEA in live mice in a non-invasive manner, we generated CIDEA reporter mice expressing multicistronic mRNAs encoding CIDEA, luciferase 2, and tdTomato proteins under the control of the *Cidea* promoter. The expression level of endogenous CIDEA protein in adipose tissue was not affected by the expression of polycistronic reporters. The two CIDEA reporters, luciferase 2 and tdTomato, correctly reflected CIDEA protein levels. Importantly, luciferase activity was induced by cold exposure and the treatment with β3-adrenergic receptor agonist CL316,243 in interscapular and inguinal adipose tissue, which was detectable by in vivo bioluminescence imaging. We further evaluated the effects of candidate brown adipogenic agents using this CIDEA reporter system and demonstrated a positive correlation between drug-induced luciferase activity and thermogenic gene expression levels both in vitro and in vivo. Collectively, we established a dual CIDEA reporter mouse model in which fluorescence and luminescence signals correctly reflect CIDEA expression, and therefore, suggested that this reporter system can be used to evaluate the thermogenic efficacy of candidate molecules.

## Introduction

The ongoing pandemic of obesity has increased the need to develop new therapeutic targets to counteract this disease and other obesity-related metabolic diseases. Obesity is characterized by abnormal accumulation of adipose tissue, and adipose tissue dysfunction is one of the risk factors associated with a high incidence of metabolic diseases^1^. However, therapeutic strategies to specifically target adipose tissue catabolic metabolism have not been fully established yet.

Adipose tissue is a major metabolic organ that can be subdivided into the following two types: white adipose tissue (WAT); and brown adipose tissue (BAT)^1,2^. WAT stores and mobilizes lipids to maintain energy homeostasis, and BAT is responsible for non-shivering thermogenesis^2^. Importantly, recent studies have demonstrated that adipocytes exhibit drastic plasticity with respect to anabolic and catabolic phenotypes^1^. Although brown and white adipocytes are known to originate from distinct mesenchymal stem cell lineages, white adipocytes can convert into brown-like adipocytes (beige adipocytes) under thermogenic stimuli, such as cold temperature^3^. This process, known as WAT browning, is characterized by the induction of uncoupling protein 1 (UCP1) expression and an increase in the mitochondrial mass and oxidative metabolism^4^.

Reduction in BAT mass and activity has been associated with aging and obesity in rodent models and humans^5^. There is accumulating evidence suggesting that interventions to enhance BAT and mitochondrial mass/function in adipocytes, such as thiazolidinedione treatment and exercise, lead to the improvement of systemic metabolic function and insulin sensitivity^6–8^. Furthermore, several anti-diabetes therapeutics, including Glucagon-like peptide 1 agonists^9^, Dipeptidyl Peptidase IV inhibitors^10^, and Sodium/glucose cotransporter 2 inhibitors^10,11^ manifest browning effects, indirectly supporting the clinical relevance and health benefits of WAT browning. Therefore, targeting the oxidative capacity and mitochondrial function of BAT has attracted attention as a potential therapeutic target. Indeed, pharmacological activation using small molecules that mimic thermogenic stimuli, such as treatment with sustained β-adrenergic receptor agonist, induces mitochondrial biogenesis and increases oxidative metabolism and thermogenesis via UCP1-dependent and -independent mechanisms^2,12^.

Drug development is a time-consuming process that requires a series of in vitro and in vivo tests and clinical trials to ensure safety and efficacy^13^. To improve the efficiency of drug development, in vivo imaging techniques have been developed as valuable tools that can provide visual information on biomarkers, treatment responses, and mechanisms of action^13^. Previously, attempts have been made to measure the thermogenic responses of adipose tissue using an in vivo UCP1 reporter system^14–17^.

Although UCP1 is a canonical brown adipocyte marker that is essential for non-shivering thermogenesis in BAT, UCP1-independent multiple mechanisms are involved in regulating energy expenditure in adipose tissue^18^. Therefore, the expression levels of genes involved in catabolic remodeling of adipose tissue with BAT-specific expression could be investigated as markers to predict the brown adipogenic/thermogenic responses of adipose tissue, in parallel to that of the UCP1 reporter systems.

Cell death-inducing DNA fragmentation factor-like effector A (CIDEA) is a lipid droplet-associated protein, which is known to regulate thermogenesis in brown/beige adipocytes^19^. Recent studies have demonstrated the transcriptional roles of CIDEA in the regulation of thermogenic gene expression, including that of UCP1^20^. The expression levels of CIDEA have been used as a brown adipocyte marker, which can be used to predict the thermogenic potential of adipocytes in relation to the regulation of energy expenditure^21^.

In the current study, to monitor the expression of CIDEA in live mice in a non-invasive manner, we generated CIDEA reporter mice expressing multicistronic mRNAs encoding CIDEA, luciferase 2 (Luc2), and tandem-dimer Tomato (tdT) proteins under the control of the *Cidea* promoter. We validated the specificity of CIDEA reporter system and evaluated the browning effects of candidate molecules that potentially induce anti-obesity effects using this CIDEA reporter system.

## Results

### Generation of CIDEA reporter mice

**Figure 1:**
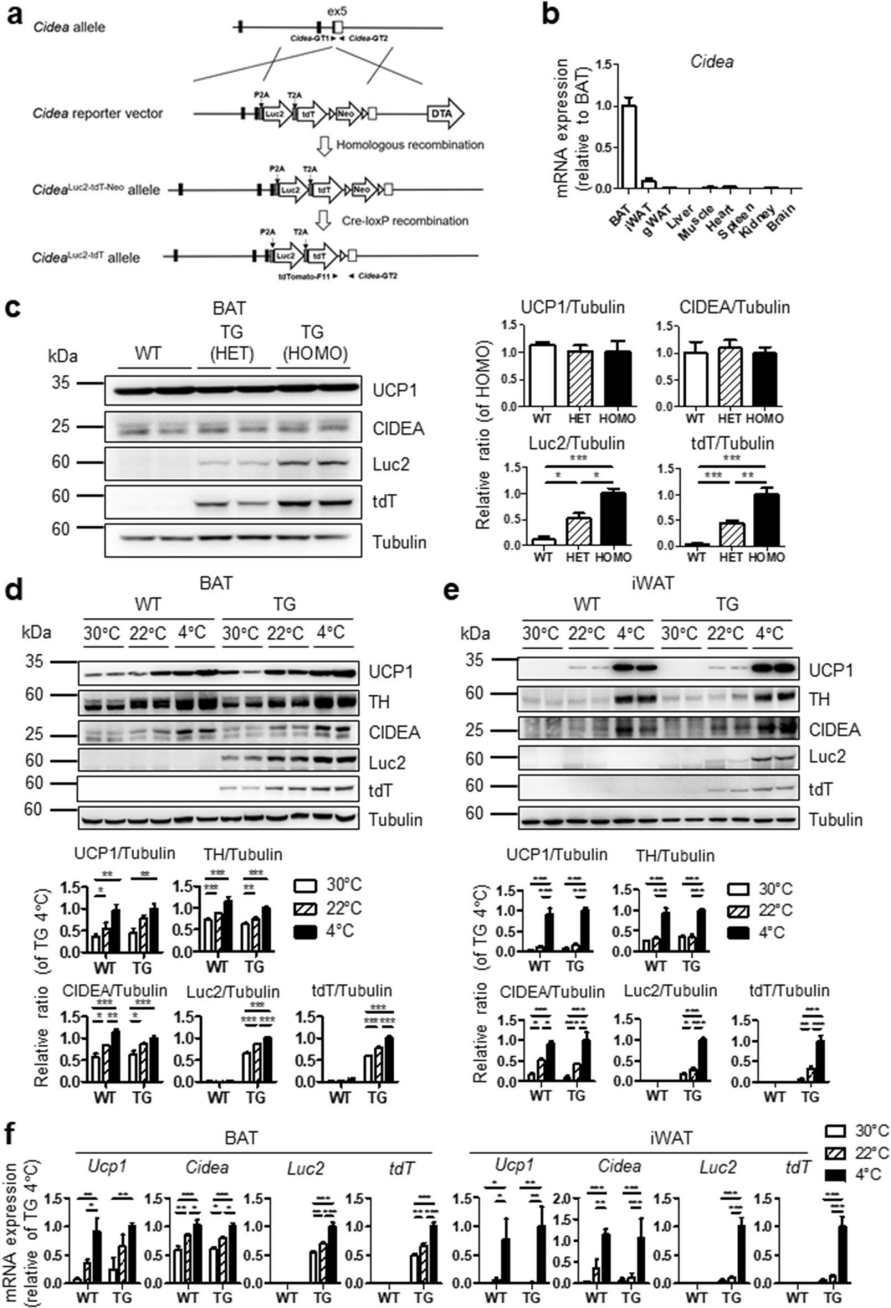
Generation of CIDEA reporter mice. (**a**) Schematic structure of the *Cidea*-P2A-Luc2-T2A-tdT (*Cidea*^Luc2-tdT^) construct. Reporter cassette encoding *Porcine teschovirus-1* 2A sequence (P2A), luciferase 2 (Luc2), *Thosea asigna virus* 2A sequence (T2A), tandem-dimer Tomato (tdT), polyadenylation signal (pA), and a positive selection marker (Neo, neomycin-resistant gene) replaced the stop codon of the *Cidea* gene. Diphtheria toxin A (DTA) gene was a negative selection marker. Genotyping primer sets for distinguishing wild type (WT) and CIDEA reporter mice were presented. (**b**) Quantitative PCR analysis of relative *Cidea* expression level in each tissue of mice (n = 6). (**c**) Immunoblot analysis of BAT of WT, heterozygous (HET), and homozygous (HOMO) CIDEA reporter mice (n = 8). (**d**–**f**) Immunoblot (**d**,**e**) and qPCR analysis (**f**) of BAT and iWAT of WT and HOMO CIDEA reporter mice (TG) maintained in thermoneutral (30 °C for 4 weeks), cold (4 °C for a week), or room temperature control condition (22 °C) (n = 4). Data were analyzed by an unpaired, two-tailed t test in (**c**) and two-way analysis of variance (ANOVA) with Bonferroni post hoc tests in (**d**–**f**) (mean ± SEM; *P < 0.05, **P < 0.01, and ***P < 0.001).

We generated a CIDEA reporter mouse line using the gene targeting method to monitor the endogenous expression of the *Cidea* gene. The reporter cassette replaced the stop codon of the *Cidea* gene (Fig. 1a). In the reporter mice, the *Cidea-*P2A-Luc2-T2A-tdT (*Cidea*^Luc2-tdT^) multicistronic transcript was produced under the control of the *Cidea* promoter, and then the translated protein was split into CIDEA, Luc2, and tdT proteins through the 2A self-cleaving peptides *Porcine teschovirus-1* 2A (P2A) and *Thosea asigna virus* 2A (T2A)^22,23^.

To examine the tissue-specific expression of CIDEA in wild type (WT) mice, we analyzed *Cidea* gene expression levels in various tissues, including adipose tissue, liver, and heart, which play an important role in lipid metabolism. Consistent with previous report^24^, *Cidea* was mainly found to be expressed in BAT (Fig. 1b).

To confirm whether the overexpression of *Cidea*^Luc2-tdT^ affects the expression levels of endogenous CIDEA expression, we analyzed CIDEA protein and mRNA expressions in BAT of WT mice, heterozygous (HET), and homozygous (HOMO) CIDEA reporter mice using the immunoblot analysis (Fig. 1c). The data indicated that there was no significant difference in CIDEA protein and mRNA levels among the three conditions (Fig. 1c, Supplementary Fig. S1). Similarly, the expression of *Cidea*^Luc2-tdT^ did not affect UCP1 protein expression in BAT (Fig. 1c). As expected, the protein expression levels of the reporter tdT and Luc2 were two-fold higher in the HOMO than in the HET mice, indicating allele dose-dependent report gene expression, whereas these reporters were not expressed in WT mice (Fig. 1c). We further tested whether changes in endogenous CIDEA expression by cold exposure (4 °C) or thermoneutrality (30 °C) are detectable in this reporter system. Immunoblot analysis of UCP1 and tyrosine hydroxylase (TH) proteins confirmed cold-induced activation and thermoneutrality-induced inactivation of BAT, respectively (Fig. 1d). Consistent with previous reports^25^ (Supplementary Fig. S2), CIDEA protein expression was cold-inducible while thermoneutrality reduced the protein levels of CIDEA in BAT and iWAT (Fig. 1d,e). Importantly, the CIDEA reporters, Luc2 and tdT correctly reflected the changes in endogenous CIDEA expression in BAT and iWAT (Fig. 1d,e). Furthermore, the cold-inducible reporter expression was validated in mRNA levels by qPCR analysis (Fig. 1f). Results indicated that mRNA expression of Luc2 and tdT mimicked cold-inducible *Cidea* mRNA expression in BAT and iWAT.

### Specificity of luminescence reporter, luciferase activity in CIDEA reporter mice

Luciferase activity assay and qPCR analysis of the tissue lysates obtained from CIDEA reporter mice confirmed that the relative luciferase activity correctly reflected *Cidea* mRNA expression level (Supplementary Fig. S3).

**Figure 2:**
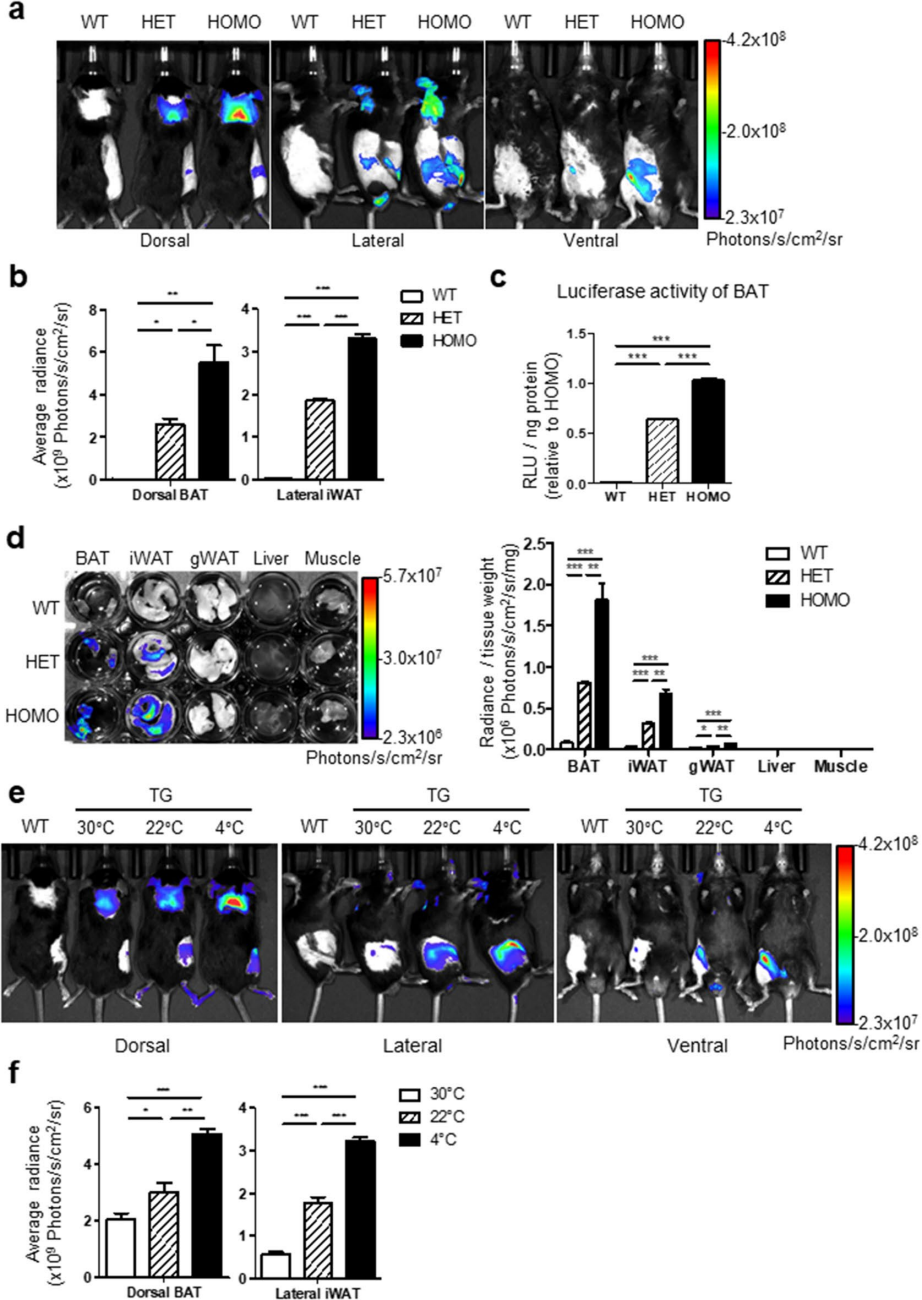
Bioluminescence indicates brown or beige adipose tissue-specific expression of CIDEA. (**a**) In vivo bioluminescence imaging of wild type (WT), heterozygous (HET), and homozygous (HOMO) CIDEA reporter mice. Mice were injected with d-luciferin (150 mg/kg, i.p.). Anatomical locations of CIDEA expression were visualized through bioluminescence. (**b**) Quantification of bioluminescence in dorsal BAT and lateral iWAT from (**a**). (**c**) Luciferase assay of BAT lysate from WT, HET, and HOMO CIDEA reporter mice. (**d**) Ex vivo bioluminescence imaging and quantification of each tissue freshly isolated from WT, HET, and HOMO mice. (**e**) In vivo bioluminescence imaging of WT and CIDEA reporter mice (TG) maintained in thermoneutral (30 °C for 4 weeks), cold (4 °C for a week), or room temperature control condition (22 °C). (**f**) Quantification of bioluminescence in dorsal BAT and lateral iWAT from (**e**) Data were analyzed by an unpaired, two-tailed t test in (**b**–**d**), (**f**) (mean ± SEM; n = 3–4, *P < 0.05, **P < 0.01, and ***P < 0.001).

To test the application of CIDEA reporter mice in non-invasive imaging, CIDEA reporter and control WT mice were injected with d-luciferin, and in vivo bioluminescence images were obtained. In dorsal position, bioluminescence signals were clearly detected in the interscapular region (Fig. 2a). In lateral images, the luminescence signals from the inguinal WAT (iWAT) were clearly visible (Fig. 2a). Quantification of luminescence signals from the images and luciferase assay of tissue lysates indicated approximately two-fold higher luminescence levels in BAT of HOMO mice than in HET mice (Fig. 2b,c). Additionally, we examined the luminescence signals in freshly isolated tissues and found that ex vivo luminescence signals were detected in BAT and iWAT (Fig. 2d,e). In vivo bioluminescence imaging detected cold-inducible CIDEA-reporter expression in BAT and iWAT (Fig. 2e,f).

### Specificity of fluorescence reporter, tdTomato expression in CIDEA reporter mice

**Figure 3:**
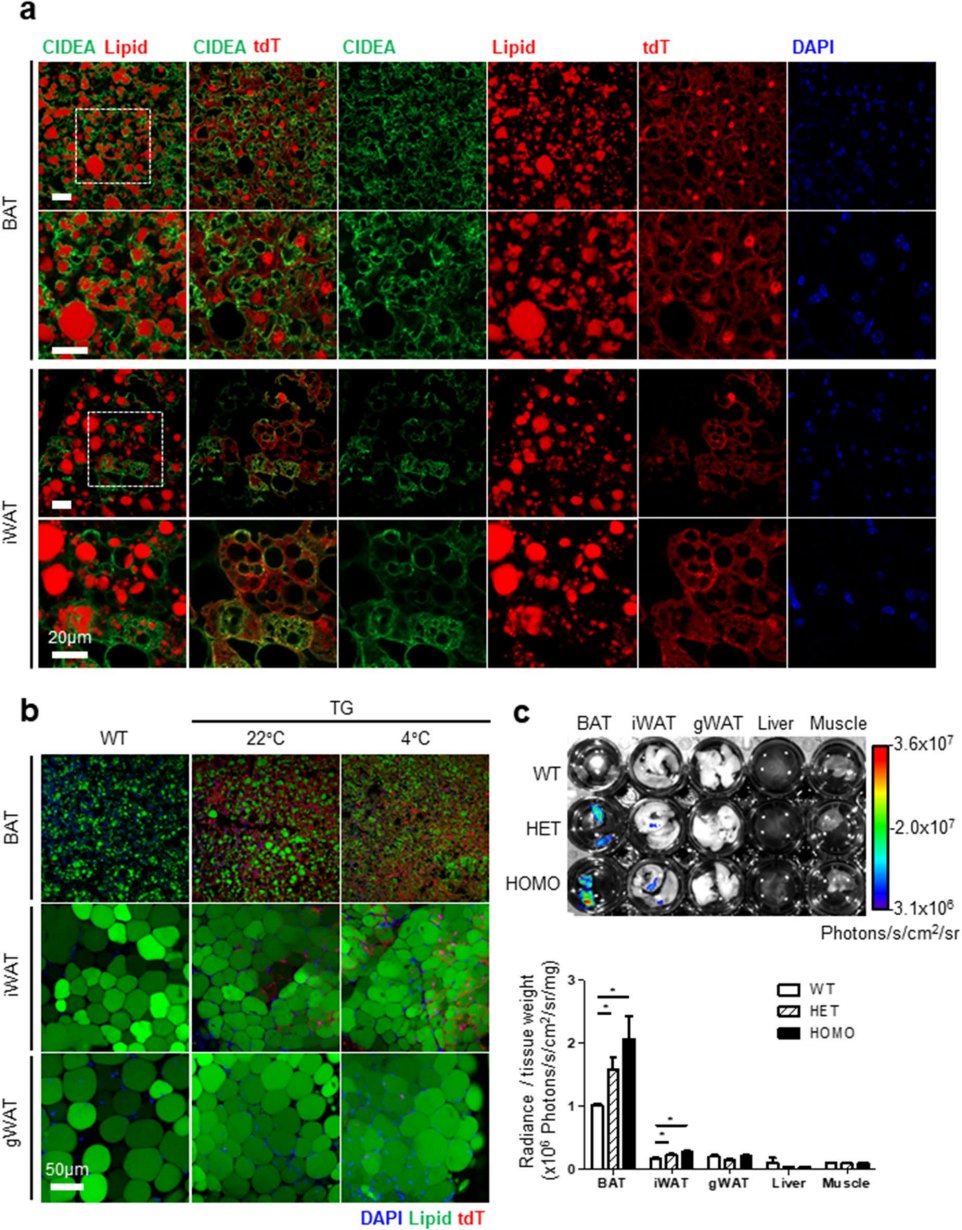
Specificity of fluorescence reporter expression in brown and beige adipose tissue of the CIDEA reporter mice. (**a**) Immunofluorescence staining of CIDEA and tdTomato in adipose tissue frozen sections of homozygous (HOMO) CIDEA reporter mice. Nuclei were counterstained with DAPI (Blue). Bar = 20 μm. (**b**) Comparison of adipose tissue frozen section (BAT) or whole mount (iWAT and gWAT) of HOMO CIDEA reporter (TG) and wild type (WT) mice. Mice were maintained in cold (4 °C for a week) or room temperature (22 °C) condition. Nuclei and lipid droplets were counterstained with DAPI and HCS LipidTox deep red. Bar = 50 μm. (**c**) Ex vivo fluorescence imaging of tissues freshly isolated from WT, Heterozygous (HET), and HOMO CIDEA reporter mice. Quantification of fluorescence signal was normalized to each tissue weight. Data were analyzed by an unpaired, two-tailed t test (mean ± SEM; n = 3 *P < 0.05).

Next, we examined the specificity of tdT expression in CIDEA reporter mice. Analysis of cryosection images indicated that all multilocular brown adipocytes in BAT expressed tdT (Fig. 3a,b). As shown by whole mount tissue imaging, tdT^+^ multilocular adipocytes were occasionally observed in iWAT, while none of adipocytes in gWAT expressed tdT (Fig. 3b). Immunohistochemical analysis of cryosections indicated that all CIDEA-expressing cells were tdT^+^ in adipose tissue and vice versa (Fig. 3a). Although the fluorescence signals from live mice were not detected by imaging, the fluorescence signals from ex vivo tissues dissected from the reporter mice were detectable and high enough to conduct quantification (Fig. 3c). Both fluorescence and bioluminescence were sufficient to reflect CIDEA expression, but only bioluminescence could be applied in a non-invasive manner.

**Figure 4:**
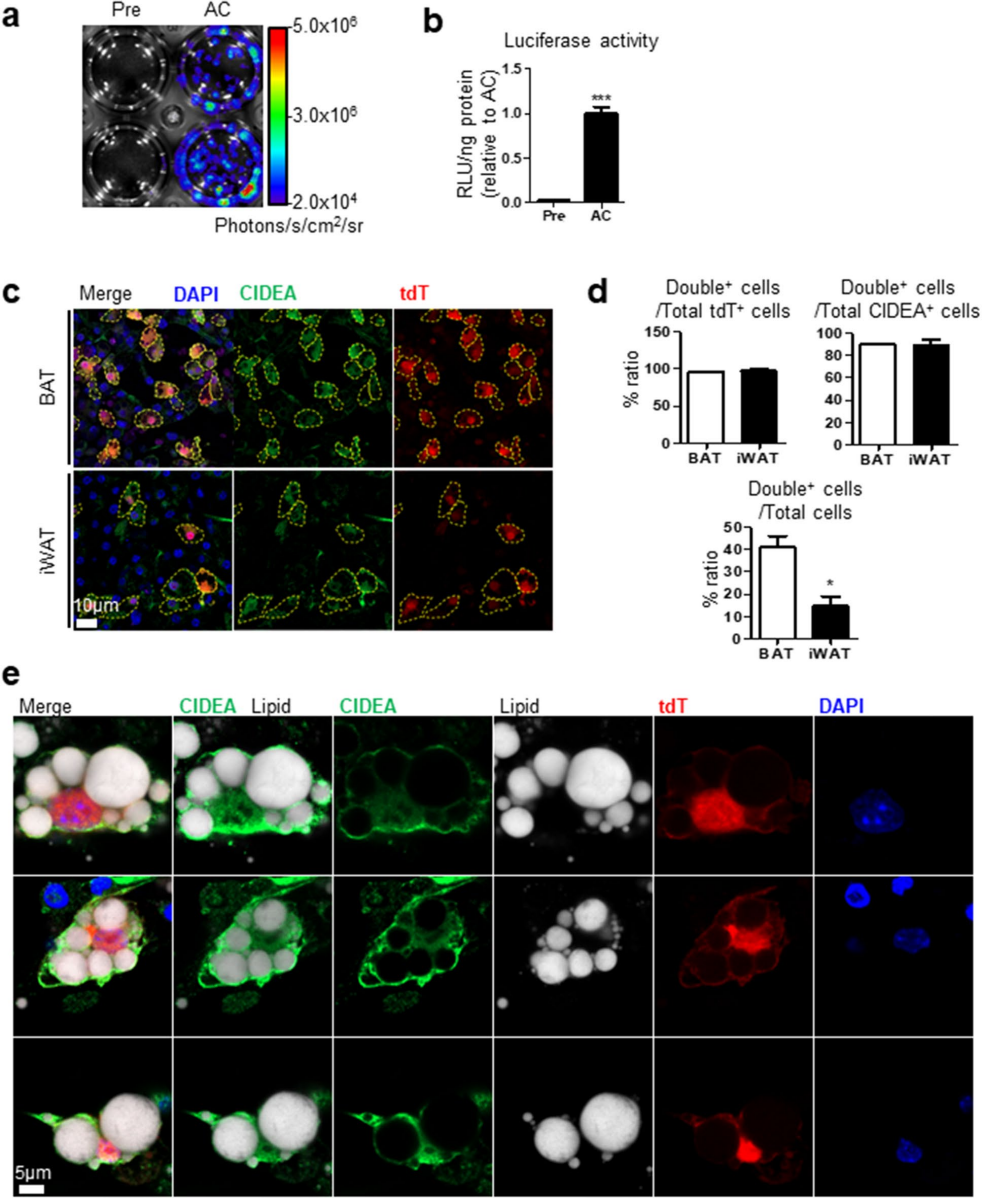
Establishment of in vitro CIDEA reporter primary culture system. Stromal vascular fractions of adipose tissue from homozygous CIDEA reporter mice were isolated, cultured, and differentiated. (**a**) In vitro bioluminescence imaging of preadipocyte (Pre) and fully differentiated adipocytes (AC) from BAT. Cells were treated with growth medium containing d-luciferin (150 μg/ml). (**b**) Luciferase assay of cell lysate from (**a**). (**c**) Immunofluorescence staining image of differentiated adipocytes from BAT and iWAT. CIDEA (Green) and tdT (Red) double positive cells (CIDEA^+^ tdT^+^) were indicated with a dashed yellow line. Nuclei were counterstained with DAPI (Blue). Bar = 10 μm. (**d**) Quantification of the number of CIDEA^+^ tdT^+^ cells per field. (**e**) High magnification images of CIDEA^+^ tdT^+^ adipocytes. Nuclei and lipid were counterstained with DAPI and HCS LipidTOX. Bar = 5 μm. Data were analyzed by an unpaired, two-tailed t test in (**b**,**d**) (mean ± SEM; n = 3–4 *P < 0.05, ***P < 0.001).

To establish an in vitro CIDEA reporter system, we used a primary culture of the stromal vascular cell from homozygous CIDEA reporter mice. Undifferentiated preadipocytes did not exhibit a luminescence signal, but differentiated brown adipocytes exhibited a strong luminescence signal (Fig. 4a,b). Also, the immunofluorescence staining image visualized the fluorescence signals of adipocytes differentiated from stromal vascular cells of BAT and iWAT (Fig. 4c–e). Similar to tissue images, CIDEA^+^ (green) cells also showed tdT signal (red) in both BAT and iWAT. As expected, CIDEA^+^ tdT^+^ cells were more abundant in BAT than iWAT (Fig. 4d).

### Application of CIDEA reporter mice for screening thermogenic drugs

**Figure 5:**
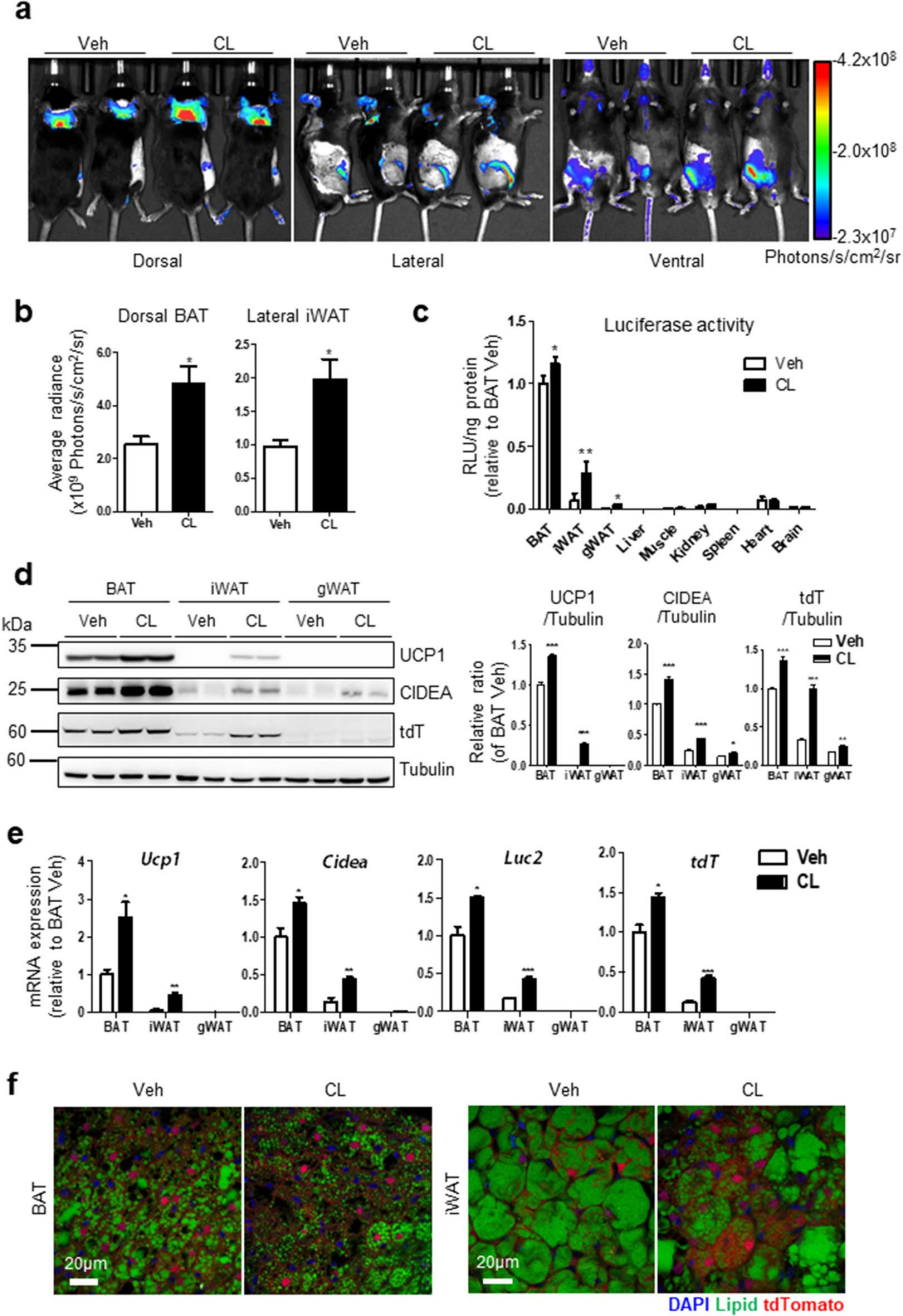
CIDEA reporter mice could reflect responsive CIDEA expression by external stimuli through luminescence and fluorescence. CIDEA reporter mice were treated with CL316,243 (CL, 1 mg/kg) or vehicles (Veh) for 3 consecutive days by intraperitoneal injection. All experiments were conducted 4 h after the last administration. (**a**) In vivo bioluminescence imaging. Mice were injected with 150 mg/kg of d-luciferin. (**b**) Quantification of bioluminescence in dorsal BAT and lateral iWAT from (**a**). (**c**) Luciferase assay of tissue lysate. (**d**,**e**) Immunoblot and qPCR analyses of each adipose tissue. (**f**) Frozen section of BAT and iWAT. Bar = 20 μm. Data were analyzed by an unpaired, two-tailed t test in (**b**–**e**) (mean ± SEM; n = 3–4, *P < 0.05, **P < 0.01, ***P < 0.001).

**Figure 6:**
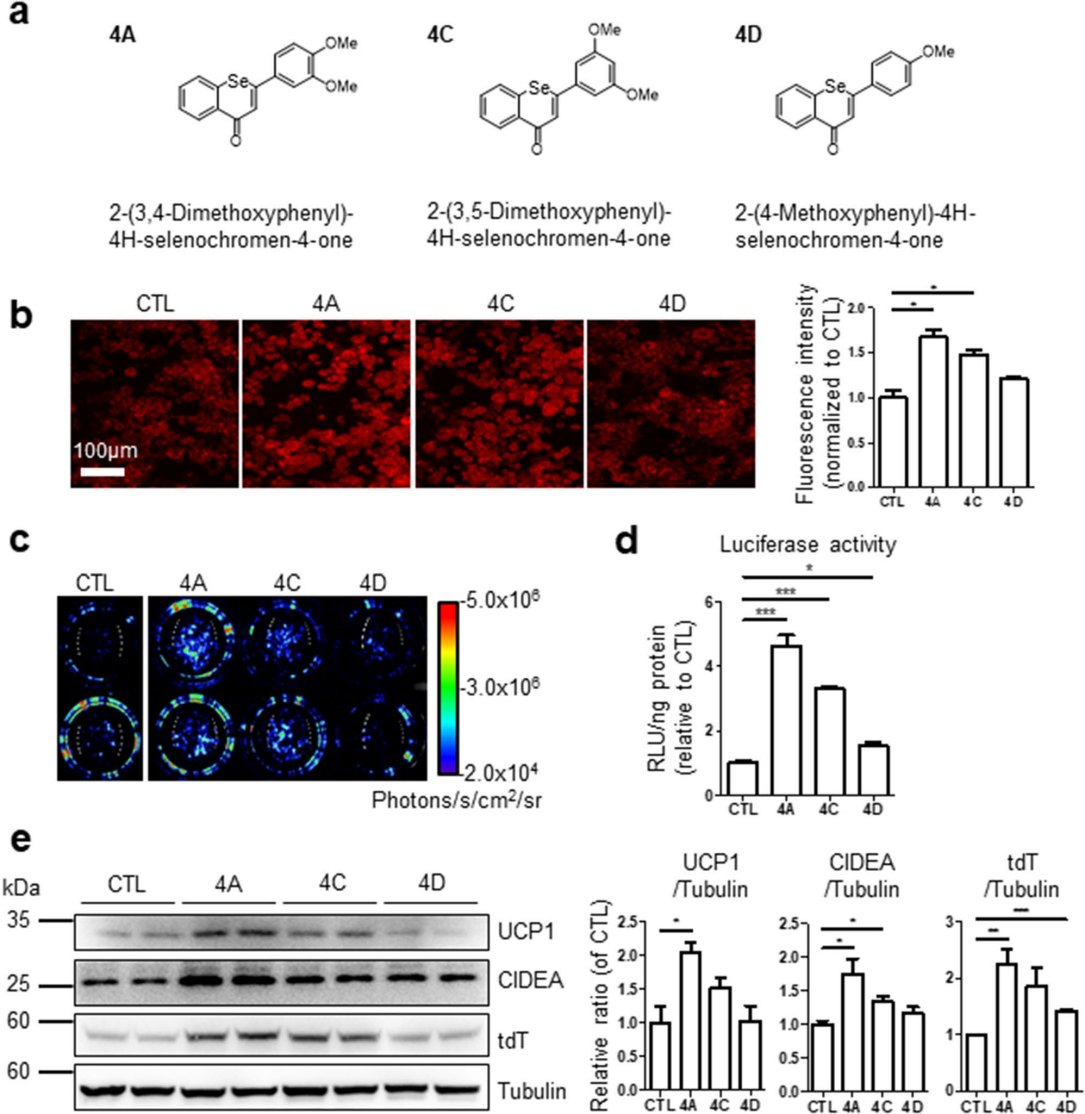
Analyses of browning effects of PMSFs by using in vitro CIDEA reporter primary culture system. Browning effects of polymethoxyselenoflavones (PMSFs) were tested utilizing in vitro CIDEA reporter primary culture system. Differentiated adipocytes from BAT were treated with the indicated compound (1 μM) for 24 h. (**a**) IUPAC name and structure of PMSFs. (**b**) Fluorescence microscope images and quantification of fluorescence intensity. (**c**) In vitro bioluminescence imaging. Cells were treated with growth medium containing d-luciferin (150 μg/ml). (**d**) Luciferase assay of cell lysate. (**e**) Immunoblot analysis and quantification. Statistical analyses were assessed with an unpaired, two-tailed t test in (**b**), (**d**–**e**) (mean ± SEM; n = 3–4, *P < 0.05, **P < 0.01, and ***P < 0.001).

To determine the application of CIDEA reporter mice in evaluating browning effects, CIDEA reporter mice were treated with the candidate thermogenic drugs. First, β3-adrenergic receptor agonist, CL316,243 (CL), was used as a positive control for assessing the browning effects. Three days of CL treatment led to an increase in the bioluminescence intensity in BAT and iWAT of the reporter mice compared to the vehicle (Fig. 5a). The bioluminescence data were consistent with the results obtained from the luciferase assay of tissue lysates (Fig. 5b,c). Western blot and qPCR analyses demonstrated that CL treatment increased CIDEA protein and mRNA levels in BAT, iWAT, and gonadal WAT (gWAT) (Fig. 5d,e). While gWAT is resistant to UCP1 induction through CL, CIDEA protein and mRNA levels were found to be induced. This suggests that CIDEA reporters are useful in detecting catabolic remodeling of visceral fat. Histological analysis showed that CL treatment led to an increase in tdT levels, especially in iWAT depot (Fig. 5f). Consequently, CIDEA reporter mice could be used for measuring the induction of CIDEA expression through assessing bioluminescence and fluorescence. To screen the candidate thermogenic drugs, we tested polymethoxyselenoflavones (PMSFs) that have been reported to exert anti-obesity effects in rodent models through the activation of lipolysis and brown adipocyte metabolism^26^. PMSFs are defined as selenocompounds generated by the substitution of an oxygen in flavone with selenium^27^. Among PMSF candidate drugs, three molecules (4A: 2-(3,4-dimethoxyphenyl)-4H-selenochromen-4-one, 4C: 2-(3,5-dimethoxyphenyl)-4H-selenochromen-4-one, and 4D: 2-(4-methoxyphenyl)-4H-selenochromen-4-one) were tested in the primary culture system (Fig. 6a). In fluorescence/luminescence analyses and western blot, PMSF 4A induced the highest CIDEA reporter expression (Fig. 6b–e), which is consistent with the browning effects measured using the immunoblot analysis of CIDEA and UCP1 (Fig. 6e), mitochondrial contents and measurement of oxygen consumption rate^26^.

**Figure 7:**
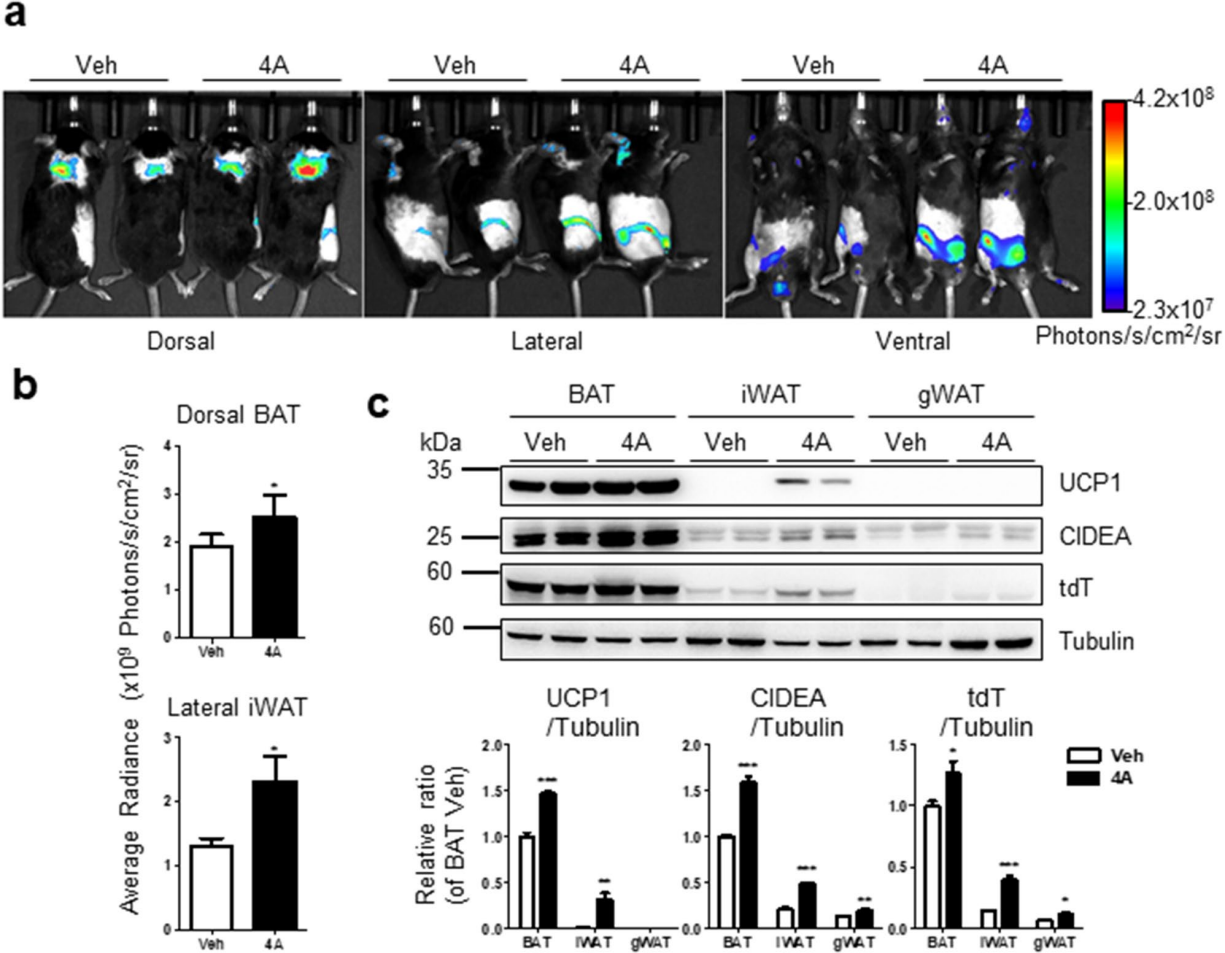
Analyses of browning effects of PMSFs by using in vivo CIDEA reporter system. To test browning effect of PMSF 4A in CIDEA reporter system, 4A (10 mg/kg, i.p.) or vehicles (Veh) were treated for 2 weeks. (**a**) In vivo bioluminescence imaging. (**b**) Quantification of bioluminescence in dorsal BAT and lateral iWAT from (**a**). (**c**) Immunoblot analysis and quantification. Statistical analyses were assessed with an unpaired, two-tailed t test (mean ± SEM; n = 4, *P < 0.05, **P < 0.01, and ***P < 0.001).

Two weeks of PMSF 4A treatment significantly led to an increase in the bioluminescence intensity in BAT and iWAT than in vehicle-treated mice (Fig. 7a,b), which was consistent with the immunoblot analysis of CIDEA expression in adipose tissue (Fig. 7c).

## Discussion

With recent advancements in the understanding of adipose tissue biology, the molecular mechanisms involved in BAT expansion and metabolic activation have been investigated as potential therapeutic targets to increase energy expenditure^28^. For instance, several candidates small molecules that induce BAT activation and phenotypic transition of WAT into BAT have been investigated in clinical trials to treat obesity and obesity-related metabolic diseases^2^. Therefore, testing the thermogenic potential of small molecules has gained attention as a tool for screening the drugs to be used as anti-obesity therapeutics.

In vivo imaging technology has been recognized as an efficient system for providing intuitively visual information on the biomarkers, treatment response, and mechanism of action^13^. It further aids the time-consuming process of candidate drug selection based on safety and efficacy^13^. Another important aspect of in vivo imaging is its non-invasiveness, which reduces efforts, such as sample collection and analysis in preclinical tests, and potentially minimizes the pain, suffering, or distress^29^.

To monitor the expression of CIDEA in live mice in a non-invasive manner, we generated CIDEA reporter mice expressing multicistronic mRNAs encoding CIDEA, Luc2, and tdT proteins under the control of the *Cidea* promoter. Protein and mRNA expression levels of endogenous CIDEA were not affected through the expression of the reporter in both heterozygous and homozygous mice. Compared to the previously developed in vivo reporter system, the advantage of this reporter system is that the endogenous expression of CIDEA is not affected by genetic modification. Another advantage of the current reporter is better sensitivity of CIDEA expression in response to the catabolic/thermogenic signals, compared to UCP1. For example, CL treatment increased CIDEA expression in gWAT, but not of UCP1. However, previous studies reported that CIDEA protein abundance is not closely correlated with mRNA expression levels in BAT of mice maintained in cold condition for 4 weeks, which might be explained by posttranscriptional regulation of CIDEA protein^30^. Therefore, although we did not test the CIDEA reporter system in long-term cold conditions, this reporter system might be more useful to monitor CIDEA gene expression in response to acute thermogenic stimuli.

We validated the correlation between *Cidea* mRNA expression and reporter luminescence signal by using multiple assays (in vivo imaging of mice, ex vivo imaging of isolated tissue, and activity assay of tissue lysates). Among those, luciferase activity of tissue lysates most closely reflected the mRNA expression levels, potentially due to technical limitations in the quantification of emitted photons from tissue in vivo or ex vivo.

In the current study, we used CIDEA as a brown adipocyte marker and biomarker to predict the thermogenic activation of adipose tissue. While CIDEA has been investigated as a lipid droplet-associated protein, the regulatory function of CIDEA in adipose tissue is not fully understood. For example, whole-body knockout mice demonstrated a lean phenotype^31^. Adipocyte-specific disruption of CIDEA is associated with the anti-diabetic phenotype through the expansion of WAT^24^. Recent work has demonstrated that the transcriptional activity of CIDEA regulates UCP1 expression^20^.

However, further studies are required to understand the role of CIDEA in catabolic lipid metabolism in adipose tissue. Nonetheless, in this study, we demonstrated that the expression level of CIDEA is correlated with UCP1 induction, mitochondrial oxidative metabolism, and thermogenic potential of adipocytes, indicating that it is a reliable system for in vivo screening of candidate thermogenic drugs.

Primary cultures obtained from adipose tissue of CIDEA reporter mice were also useful for detecting the thermogenic potentials using the dual reporters, Luc2 and tdT expression, whose values were positively correlated with the mitochondrial oxygen consumption rates of adipocytes. Comparison or concomitant use with UCP1 reporters may help improve the screening efficiency and validity of the system for the identification of potential thermogenic drugs.

In summary, we established CIDEA reporter mouse model for non-invasive imaging of adipose tissue catabolic remodeling. The data indicated that the levels of luciferase activity and fluorescence signals were positively correlated with thermogenic gene expression levels and mitochondrial oxidative metabolism both in vitro and in vivo. Therefore, this CIDEA reporter system acts as a convenient model for the identification of potential thermogenic drugs.

## Materials and methods

### Generation of CIDEA reporter mice

A BAC clone (129S7/AB2.2 library, bMQ377H14) possessing the *Cidea* locus was obtained from Source BioScience (UK). The *Cidea* reporter vector was constructed using a recombineering system^32^ (Fig. 1a). A 6.7-kb genomic DNA fragment of *Cidea* was retrieved from BAC DNA and inserted into pLMJ235 possessing the diphtheria toxin A (DTA) gene as a negative selection marker. The P2A-Luc2-T2A-tdT-pA-Neo reporter cassette including P2A, Luc2, T2A, tdT, polyadenylation signal (pA), and a positive selection marker (Neo, neomycin-resistant gene) replaced the stop codon of the *Cidea* gene (Supplementary Fig. S4a). The *Cidea* reporter vector was linearized with NotI and electroporated into 2 × 10^7^ J1 mouse embryonic stem cells (ESCs). Approximately 300 neomycin-resistant ESC colonies were screened by Southern blot analysis using the probe (Supplementary Fig. S4b). Male chimeras obtained from correctly targeted ESCs were bred with C57BL/6N females to establish the *Cidea*^Luc2-tdT-Neo^ mouse strain. *Cidea*^Luc2-tdT-Neo^ mice were crossed with β-actin-Cre mice (Jackson Laboratory) to remove the neomycin-resistant gene (Neo) flanked by the loxP sequences, which generated the CIDEA reporter (*Cidea*^Luc2-tdT^) mouse strain.

### Animal experiments

All of the protocols related to animal experiments were approved by the Institutional Animal Care and Use Committees of Gachon University (permission number LCDI-2015-0032; LCDI-2016-0084) and Seoul National University (permission number SNU-191113-2; SNU-200904-7-3) and conducted according to the committee’s guidelines, and the study was carried out in compliance with the ARRIVE guidelines. Mice were kept on a 12-h light/12-h dark cycle and fed with free access to food (standard chow diet) and water in a temperature (22 ± 1 °C) and humidity (55 ± 5%) controlled room. All experiments were performed using 6 to 8-week-old male mice, and all analyses were carried out by researchers blinded to the group allocation. For thermoneutral conditions, mice originally housed at room temperature (22 ± 1 °C) were transferred to constant climate chamber (Memmert) and maintained at 30 °C for 4 weeks. For cold conditions, mice were maintained at 4 °C for a week. For β3-adrenergic receptor activation, mice were treated with 1 mg/kg/day of CL316,243 (Sigma) for 3 days. To test the browning effect of polymethoxyselenoflavone (PMSF) 4A, 10 mg/kg/day of 4A was intraperitoneally injected to CIDEA reporter mice for 2 weeks described like previously^26^.

### Genotyping

CIDEA reporter mice were genotyped by PCR analysis of genomic DNA isolated from tails. Tails were lysed alkaline lysis reagent (containing 25 mM NaOH and 0.2 mM Sodium EDTA; pH 12), heated at 98 °C for 1 h, and neutralized with neutralization reagent (containing 40 mM Tris–HCl; pH 5). The supernatant containing genomic DNA and MyTaq HS Red Mix (Bioline) were used for PCR. Genotyping primer sequences were described in Supplementary Table S1. The PCR products were 200 bp for WT or 299 bp for transgene allele, respectively.

### Gene expression analysis

Quantitative PCR analyses were performed as described previously^33^. Briefly, total RNA was isolated using TRIzol reagent (Invitrogen) according to the manufacturer’s instructions. High Capacity cDNA Reverse Transcription kit (Applied Biosystems) was used to synthesize cDNA. iQ SYBR^®^ Green Supermix (Bio-Rad) was used for quantitative PCR reactions. Relative expression levels of each gene were calculated using the 2-ΔCt method. Peptidylprolyl Isomerase A (PPIA) was used for the normalization. qPCR primer sequences were described in Supplementary Table S2.

### Western blot analyses

Western blot analyses were performed as described previously^33^. Briefly, cells were lysed using RIPA buffer (Invitrogen) containing SIGMAFAST Protease Inhibitor Cocktail (Sigma) and PhoSTOP phosphatase inhibitors (Roche). Adipose tissue lysates were prepared using PRO-PREP Protein Extraction Solution (iNtRON Biotechnology). Primary antibodies used for western blot analyses were described in Supplementary Tables S3. NIH ImageJ software was used for the quantification. Full-length immunoblot membrane images are attached in Supplementary Information.

### Immunofluorescence staining and confocal microscopy

Immunofluorescence staining was performed as described previously^33^. CIDEA antibody (Rabbit, 1:100, Novus Biologicals, RRID: AB_11012002) was used for immunofluorescence detection. Goat anti-rabbit-Alexa Fluor 488 (1:500, Invitrogen, RRID: AB_143165) was used as a secondary antibody. Normal rabbit IgG control (Santa Cruz) or the omission of the primary antibody was used as a negative control. DAPI (Sigma) and HCS LipidTOX deep red (Invitrogen) were used for nucleus and lipid droplet counterstaining. Stained slides were imaged on a confocal laser-scanning microscope (Zeiss LSM 800 or Leica TCS SP8).

### Luciferase assay

Luciferase activity of tissue or cell lysate was analyzed using Luciferase Assay System (Promega, E4030) with a microplate luminometer (Centro LB 960, BERTHOLD), according to the manufacturer’s instructions. Briefly, tissues or cells were lysed in Reporter Lysis Buffer, and centrifuged at 10,000 x *g* for 10 min at 4 °C, and the supernatant was used for the analysis^34^. 10 μl of each sample was loaded on 96-well white-bottom microplates (Thermo), and then 50 μl of Luciferase Assay Solution was added for luciferase activity measurement. Luciferase activity values were normalized to the protein concentration of lysate.

### Bioluminescence and fluorescence imaging

In vivo bioluminescence was visualized using an optical imaging device (Ami-X imaging systems, Spectral Instruments Imaging). The mouse hair covering the regions of interest was removed prior to imaging. Mice were anesthetized with isoflurane gas (5% for induction and 2% for maintenance). To detect bioluminescence signal, mice were given d-luciferin (Goldbio, 150 mg/kg) by intraperitoneal injection, and imaged 10 min after the injection. Images were quantified by the Aura Software (Version 2.2.1.1, Spectral Instruments Imaging).

For ex vivo analysis, tissues were obtained from the CIDEA reporter mice treated with d-luciferin (150 mg/kg, i.p.). The freshly isolated tissues were placed in 24 well plates and maintained in PBS containing d-luciferin (300 μg/ml) during imaging. For in vitro analysis, cells were maintained in DMEM containing d-luciferin (150 μg/ml) during imaging.

To detect ex vivo and in vitro fluorescence signals, tdT was excited with 570 nm light source and detected with 610 nm emission filter.

### Primary cell culture

Adipose tissues obtained from CIDEA reporter mice were processed for stromal vascular fraction isolation as previously described^35^. For adipogenic differentiation, confluent cells were exposed to differentiation medium: growth medium (DMEM with 10% FBS and 1% penicillin–streptomycin) containing 2.5 mM of isobutylmethylxanthine (Cayman), 1 μM of dexamethasone (Cayman), 1 μg/ml of insulin (Sigma), 0.125 mM of indomethacin (Cayman), and 1 nM of triiodothyronine (T3, Cayman) for 3 days, and then exposed to maintenance medium: growth medium containing 1 μg/ml of insulin and 1 nM of T3 for 3 days. Differentiated adipocytes were treated with PMSFs (1 μM) in growth medium for 24 h. Zeiss LSM800 confocal microscope or Nikon Ts2-FL fluorescence microscope was used to detect fluorescence signals.

### Statistical analysis

GraphPad Prism 5 (GraphPad Software, La Jolla, CA, USA) was utilized for statistical analysis. Data were presented as mean ± standard errors of the mean (SEM). Statistical significance between two groups was determined by an unpaired t test. Comparisons among multiple groups were performed using a two-way analysis of variance (ANOVA), with Bonferroni post hoc tests to determine P values.

## Supplementary Information

Supplementary Information 1.

## Data Availability

All data generated or analyzed during this study are included in this published article and its Supplementary Information.

## References

[1] Lee YH, Mottillo EP, Granneman JG (2014). Adipose tissue plasticity from WAT to BAT and in between. Biochem. Biophys. Acta..

[2] Kajimura S, Spiegelman BM, Seale P (2015). Brown and Beige fat: Physiological roles beyond heat generation. Cell Metab..

[3] Ding C (2018). De novo reconstruction of human adipose transcriptome reveals conserved lncRNAs as regulators of brown adipogenesis. Nat. Commun..

[4] Boutant M (2017). Mfn2 is critical for brown adipose tissue thermogenic function. EMBO J..

[5] Zoico E (2019). Brown and Beige adipose tissue and aging. Front. Endocrinol. (Lausanne).

[6] Menshikova EV (2006). Effects of exercise on mitochondrial content and function in aging human skeletal muscle. J. Gerontol. A Biol. Sci. Med. Sci..

[7] Farmer SR (2008). Molecular determinants of brown adipocyte formation and function. Genes Dev..

[8] Bogacka I, Xie H, Bray GA, Smith SR (2005). Pioglitazone induces mitochondrial biogenesis in human subcutaneous adipose tissue in vivo. Diabetes.

[9] Beiroa D (2014). GLP-1 agonism stimulates brown adipose tissue thermogenesis and browning through hypothalamic AMPK. Diabetes.

[10] Takeda K (2018). The dipeptidyl peptidase-4 (DPP-4) inhibitor teneligliptin enhances brown adipose tissue function, thereby preventing obesity in mice. FEBS Open Bio.

[11] Xu L (2017). SGLT2 inhibition by empagliflozin promotes fat utilization and browning and attenuates inflammation and insulin resistance by polarizing M2 macrophages in diet-induced obese mice. EBioMedicine.

[12] Flachs P, Rossmeisl M, Kuda O, Kopecky J (2013). Stimulation of mitochondrial oxidative capacity in white fat independent of UCP1: A key to lean phenotype. Biochim. Biophys. Acta Mol. Cell Biol. Lip..

[13] Willmann JK, van Bruggen N, Dinkelborg LM, Gambhir SS (2008). Molecular imaging in drug development. Nat. Rev. Drug Discov..

[14] Galmozzi A (2014). ThermoMouse: An in vivo model to identify modulators of UCP1 expression in brown adipose tissue. Cell Rep..

[15] Mao L (2017). Visualization and quantification of browning using a *Ucp1*-2A-luciferase knock-in mouse model. Diabetes.

[16] Wang H (2019). A dual Ucp1 reporter mouse model for imaging and quantitation of brown and brite fat recruitment. Mol. Metab..

[17] Fukuda A (2019). Non-invasive in vivo imaging of UCP1 expression in live mice via near-infrared fluorescent protein iRFP720. PLoS One.

[18] Roesler A, Kazak L (2020). UCP1-independent thermogenesis. Biochem. J..

[19] Barneda D (2015). The brown adipocyte protein CIDEA promotes lipid droplet fusion via a phosphatidic acid-binding amphipathic helix. Elife.

[20] Jash S, Banerjee S, Lee M-J, Farmer SR, Puri V (2019). CIDEA transcriptionally regulates UCP1 for britening and thermogenesis in human fat cells. iScience.

[21] Seale P (2007). Transcriptional control of brown fat determination by PRDM16. Cell Metab..

[22] Donnelly MLL (2001). Analysis of the aphthovirus 2A/2B polyprotein 'cleavage' mechanism indicates not a proteolytic reaction, but a novel translational effect: A putative ribosomal 'skip'. J. Gen. Virol..

[23] Doronina VA (2008). Site-specific release of nascent chains from ribosomes at a sense codon. Mol. Cell. Biol..

[24] Abreu-Vieira G (2015). Cidea improves the metabolic profile through expansion of adipose tissue. Nat. Commun..

[25] Roh HC (2018). Warming induces significant reprogramming of Beige, but not Brown, adipocyte cellular identity. Cell Metab..

[26] Kwon H-J (2020). Polymethoxyselenoflavones exert anti-obesity effects through activation of lipolysis and brown adipocyte metabolism. Int. J. Obes..

[27] Choi Y-S (2015). Synthesis and evaluation of neuroprotective selenoflavanones. Int. J. Mol. Sci..

[28] Lee Y-H, Jung Y-S, Choi D (2014). Recent advance in brown adipose physiology and its therapeutic potential. Exp. Mol. Med..

[29] Marzola P, Boschi F, Moneta F, Sbarbati A, Zancanaro C (2016). Preclinical in vivo imaging for fat tissue identification, quantification, and functional characterization. Front. Pharmacol..

[30] Fischer AW (2017). UCP1 inhibition in Cidea-overexpressing mice is physiologically counteracted by brown adipose tissue hyperrecruitment. Am. J. Physiol. Endocrinol. Metab..

[31] Zhou Z (2003). Cidea-deficient mice have lean phenotype and are resistant to obesity. Nat. Genet..

[32] Warming S, Costantino N, Court D, Jenkins N, Copeland NJNAR (2005). Simple and highly efficient BAC recombineering using galK selection. Nucleic Acids Res..

[33] Lee YH, Kim SN, Kwon HJ, Granneman JG (2017). Metabolic heterogeneity of activated beige/brite adipocytes in inguinal adipose tissue. Sci. Rep..

[34] MarstonManthorpe FC-J, JukkaHartikka JF, Ann R, Michal M, Varavani D (1993). Gene therapy by intramuscular injection of plasmid DNA: Studies on firefly luciferase gene expression in mice. Human Gene Ther..

[35] Lee YH, Petkova AP, Mottillo EP, Granneman JG (2012). In vivo identification of bipotential adipocyte progenitors recruited by beta3-adrenoceptor activation and high-fat feeding. Cell Metab..

