# Supplementary material for: Development of CIDEA reporter mouse model and its application for screening thermogenic drugs
Source: Sci Rep. 2021 Sep 16;11:18429. doi: 10.1038/s41598-021-97959-0 (PMC8445935; doi:10.1038/s41598-021-97959-0)

**Supplementary Information**

**Development of CIDEA reporter mouse model and its application for screening thermogenic drugs**

Yeonho Son^1#^, Cheoljun Choi^1#^, Cheol Song^2^, Hyeonyeong Im^1^, Yoon Keun Cho^1^, Ju Seung Son^1^, Sungug Joo^1^, Yoonjoe Joh^1^, Young Jae Lee^2,3*^, Je Kyung Seong^4*^, and Yun-Hee Lee^1*^,

^1^College of Pharmacy and Research Institute of Pharmaceutical Sciences, Seoul National University, Seoul 08826, Republic of Korea

^2^Korea Mouse Phenotyping Center (KMPC) and Lee Gil Ya Cancer and Diabetes Institute, Gachon University, Incheon, Republic of Korea

^3^Department of Biochemistry, College of Medicine, Gachon University, Incheon, Republic of Korea

^4^Korea Mouse Phenotyping Center (KMPC), Seoul National University, Seoul, Republic of Korea

#These authors contributed equally to this work.

***Correspondence**

**Yun-Hee Lee**

College of Pharmacy and Research Institute of Pharmaceutical Sciences, Seoul National University, 29-Room # 311,1 Gwanak-ro, Gwanak-gu, Seoul, 08826, Republic of Korea

Tel: 82-2-880-2139, Fax: 82-2-872-1795, e-mail: yunhee.lee@snu.ac.kr

**Je Kyung Seong**

Korea Mouse Phenotyping Center (KMPC), Seoul National University, Seoul, Republic of Korea

e-mail: snumouse@snu.ac.kr

**Young Jae Lee**

Department of Biochemistry, College of Medicine, Gachon University, Incheon, Republic of Korea

Tel: +82-32-899-6590, Fax: +82-32-899-6591, e-mail: leeyj@gachon.ac.kr

**Supplementary Figures**

**
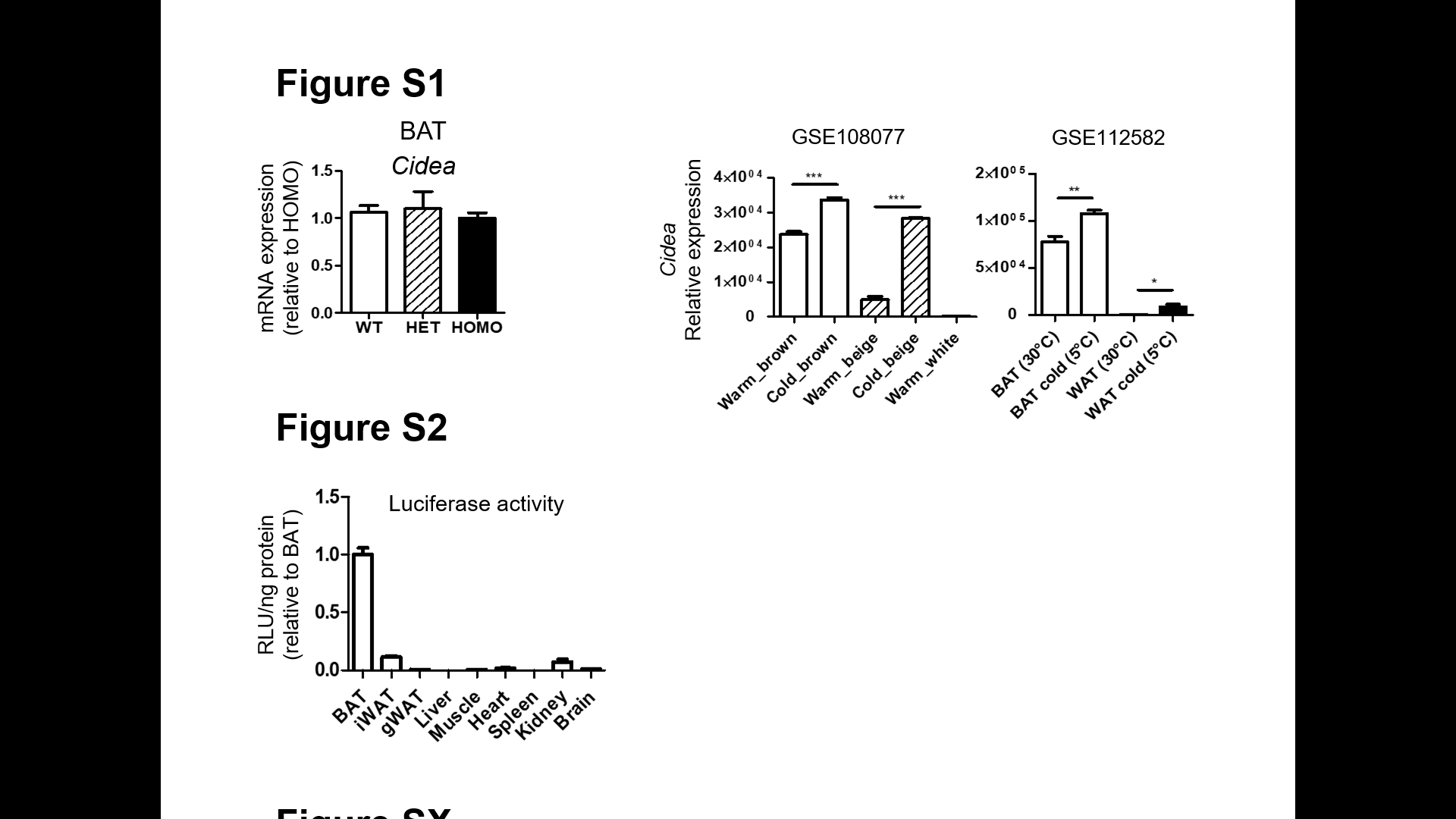
**

**Supplementary Figure S1. Comparison of *Cidea* mRNA expression in BAT of Wild type and CIDEA reporter mice.** Quantitative PCR analysis of relative *Cidea* expression level in BAT of Wild type (WT), Heterozygous (HET), and Homozygous (HOMO) CIDEA reporter mice. Statistical analyses were assessed with an unpaired, two-tailed t-test (mean ± SEM; n = 4).

**
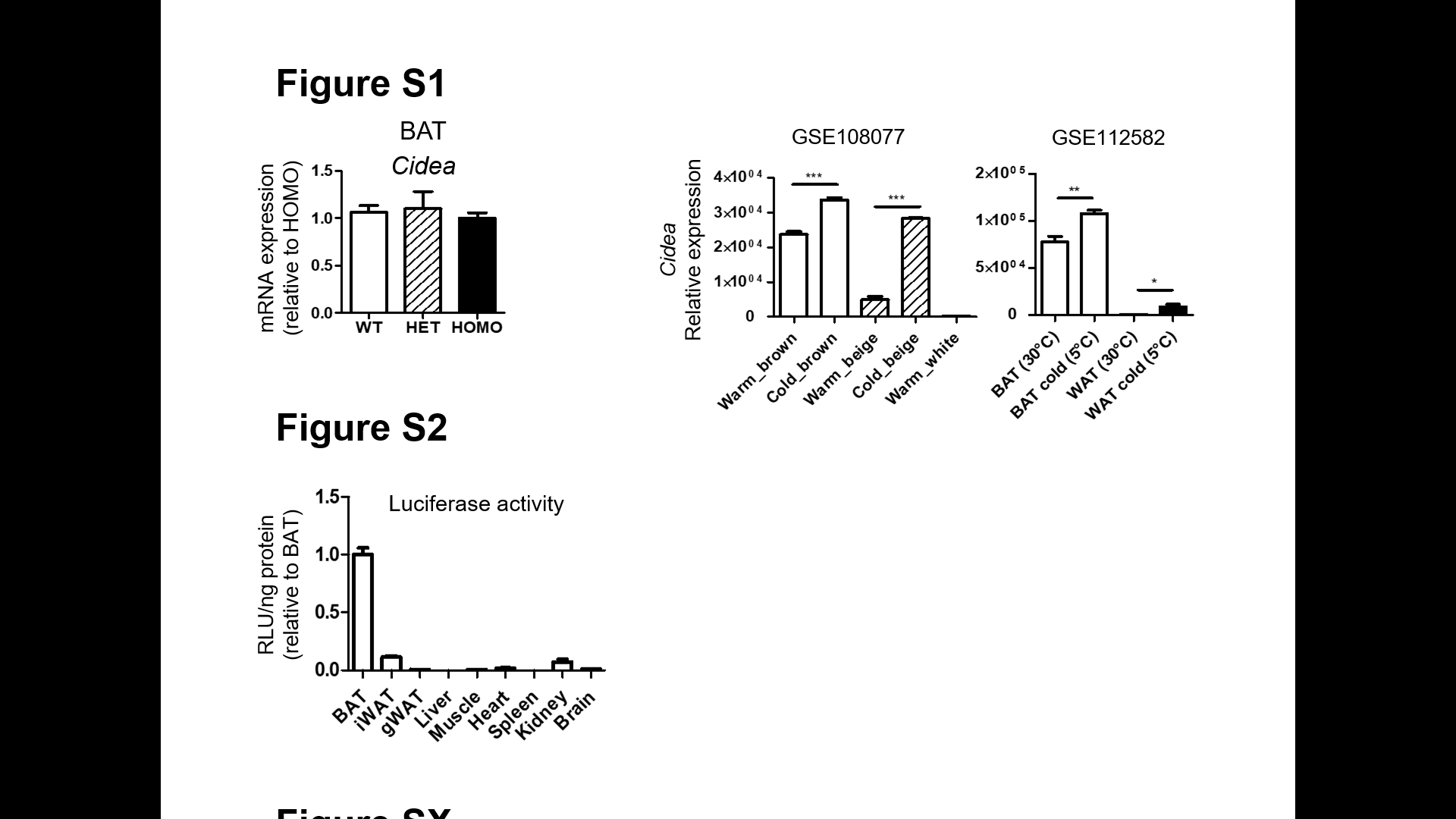
**

**Supplementary Figure S2. Transcript expression analysis of Cidea in adipose tissue.** Transcript expression of Cidea was analyzed in brown, beige, and white adipose tissue from mice exposed to warm or cold temperature. The data were determined by publicly available transcriptomic analysis: Gene Expression Omnibus (GEO) repository, accession number GSE108077 and GSE112582. Statistical analyses were assessed with an unpaired, two-tailed t-test (mean ± SEM; n = 3-5).


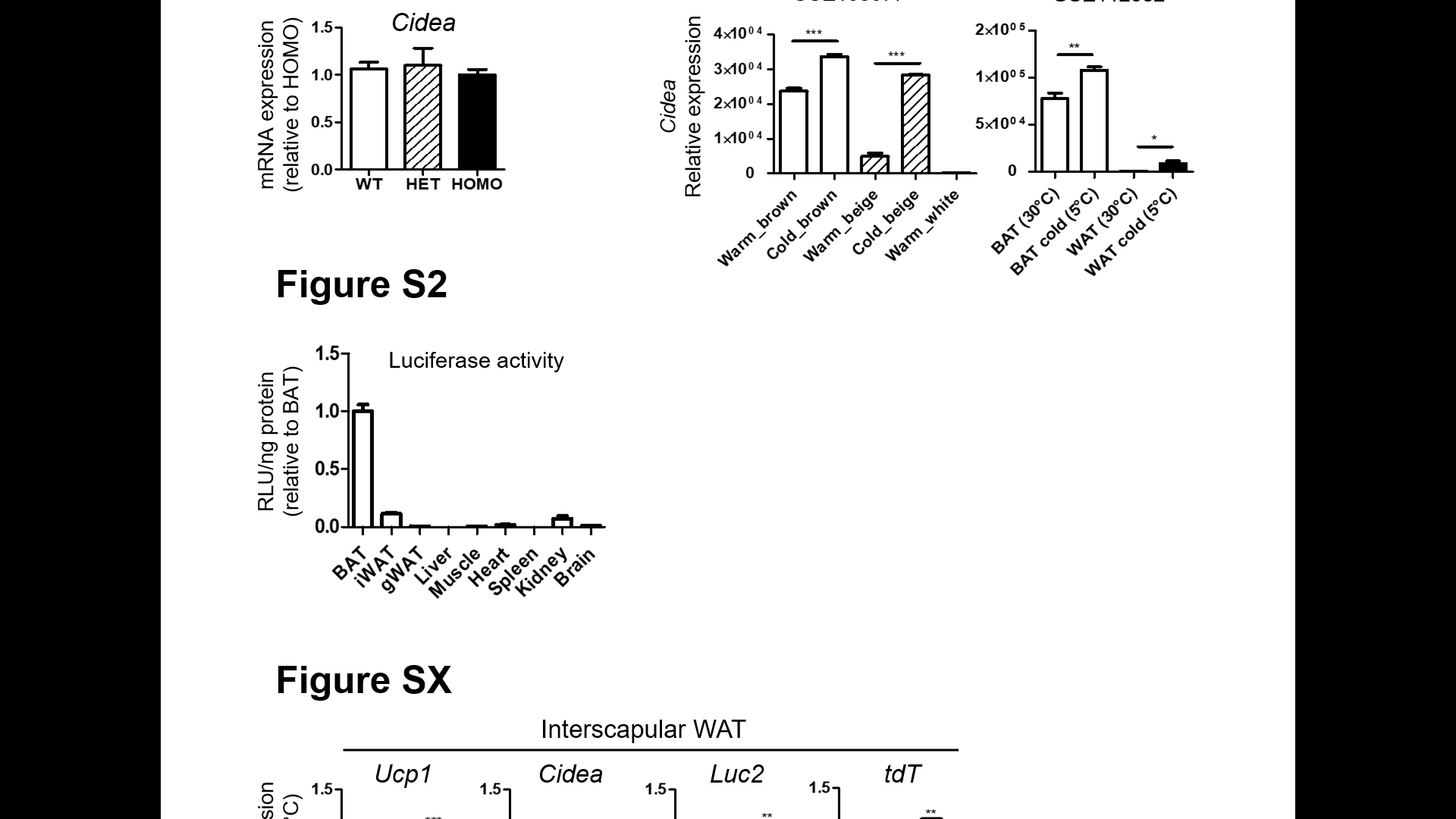


**Supplementary Figure S3. Luciferase assay of various tissue from CIDEA reporter mice.** CIDEA expression in various tissue was evaluated by Luciferase assay of tissue lysate from Homozygous CIDEA reporter mice (mean ± SEM; n = 4) (related to Fig. 1B).

**
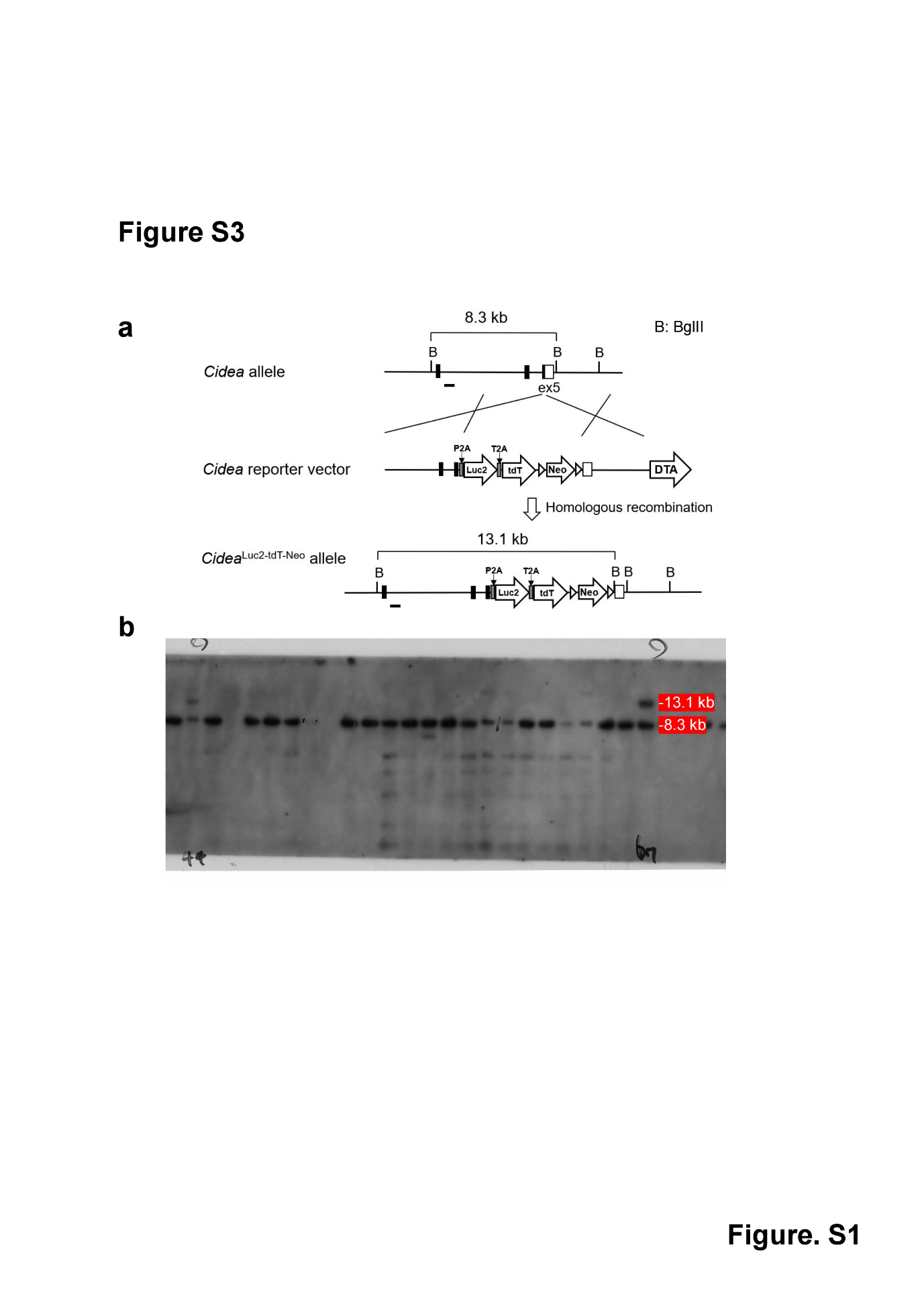
**

**Supplementary Figure S4. Generation of CIDEA reporter mice.**

Introduction of *Cidea* reporter cassette (related to Fig. 1a). (**a**) *Cidea* reporter cassette was introduced by homologous recombination. (**b**) Southern blot analysis for selection of neomycin-resistant colonies. Positive colonies could be distinguished using sequence digested with BglII. Wild type is up to 8.3 kb and CIDEA reporter allele is up to 13.1 kb.

**Supplementary Tables**

**Supplementary Table S1. Genotyping primers**

| **Target** | **Forward (5΄→3΄)** | **Reverse (5΄→3΄)** |
| --- | --- | --- |
| Wild type | *Cidea-*GT1:  TGC AGG AAT CTG CTG AGG TTT ATG | *Cidea-*GT2:  CCA CAG CCT ATA ACA GAG AGC AGG |
| Transgene  (*Cidea*^Luc2-tdT^) | tdTomato-F11:  TAC GGC ATG GAC GAG CTG TAC AAG | *Cidea-*GT2:  CCA CAG CCT ATA ACA GAG AGC AGG |

**Supplementary Table S2. qPCR primers**

| **Genes** | **Forward (5΄→3΄)** | **Reverse (5΄→3΄)** |
| --- | --- | --- |
| *Ucp1*  *Cidea*  *Luc2*  *tdT* | TGG CCT CTC AGT GGA TGT G  TGC TCT TCT GTA TCG CCC AGT  TAA GGT GGT GGA CTT GGA CA  GTG ACC GTG ACC CAG GAC | CGT GGT CTC CCA GCA TAG AAG  GCC GTG TTA AGG AAT CTG CTG  GTT GTT AAC GTA GCC GCT CA  CGC GCA TCT TCA CCT TGT |
| *Ppia* | GTG GTC TTT GGG AAG GTG AA | TTA CAG GAC ATT GCG AGC AG |

**Supplementary Table S3. Primary antibodies used for western blots**

| **Antibody** | **Host** | **Manufacturer** | **Catalog #** | **RRIDs** | **Dilution** |
| --- | --- | --- | --- | --- | --- |
| UCP1 | Rabbit | Cell signaling | UCP11-A | AB_1624298 | 1:2000 |
| CIDEA  Luciferase  DsRed  (for tdTomato) | Rabbit  Mouse  Rabbit | Novus Biologicals  Santa Cruz  Takara | NBP1-76950  sc-74548  632496 | AB_11012002  AB_1125118  AB_10013483 | 1:1000  1:1000  1:1000 |
| Tyrosine Hyroxylase | Rabbit | Cell signaling | 2792 | AB_2303165 | 1:1000 |
| Tubulin | Rabbit | Cell signaling | 2148 | AB_2288042 | 1:1000 |


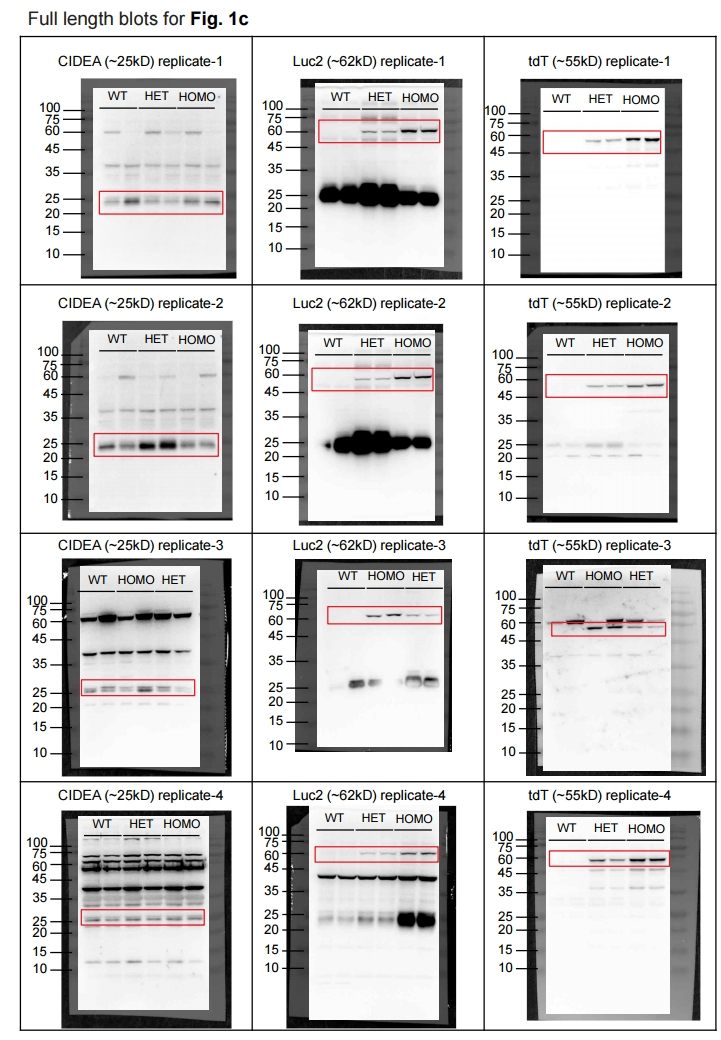

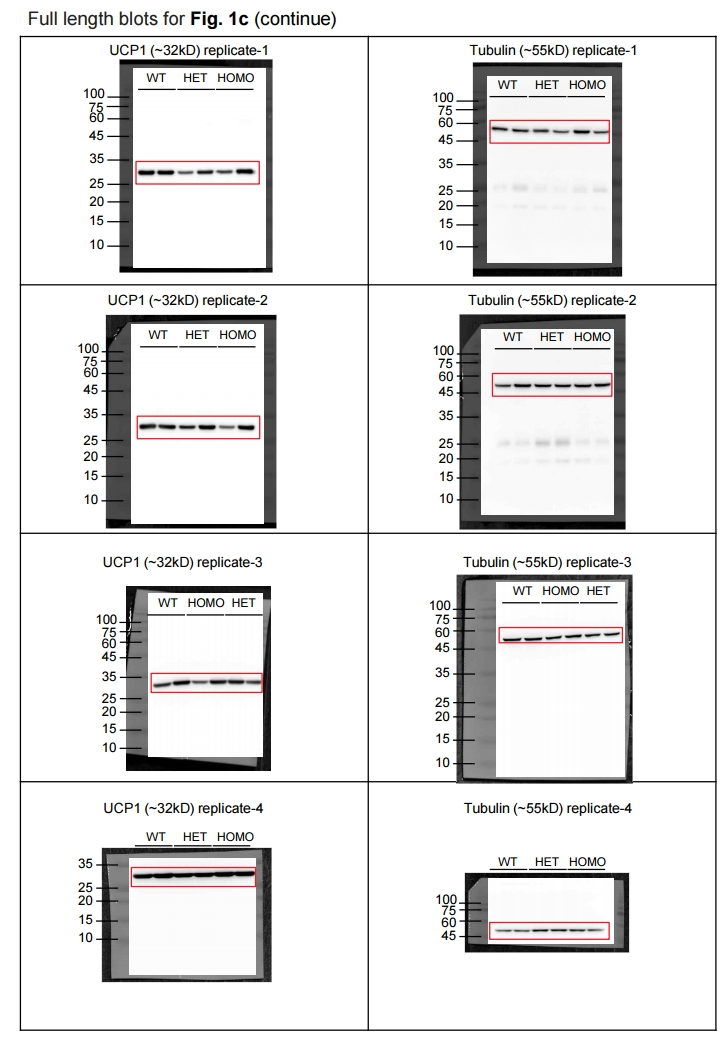

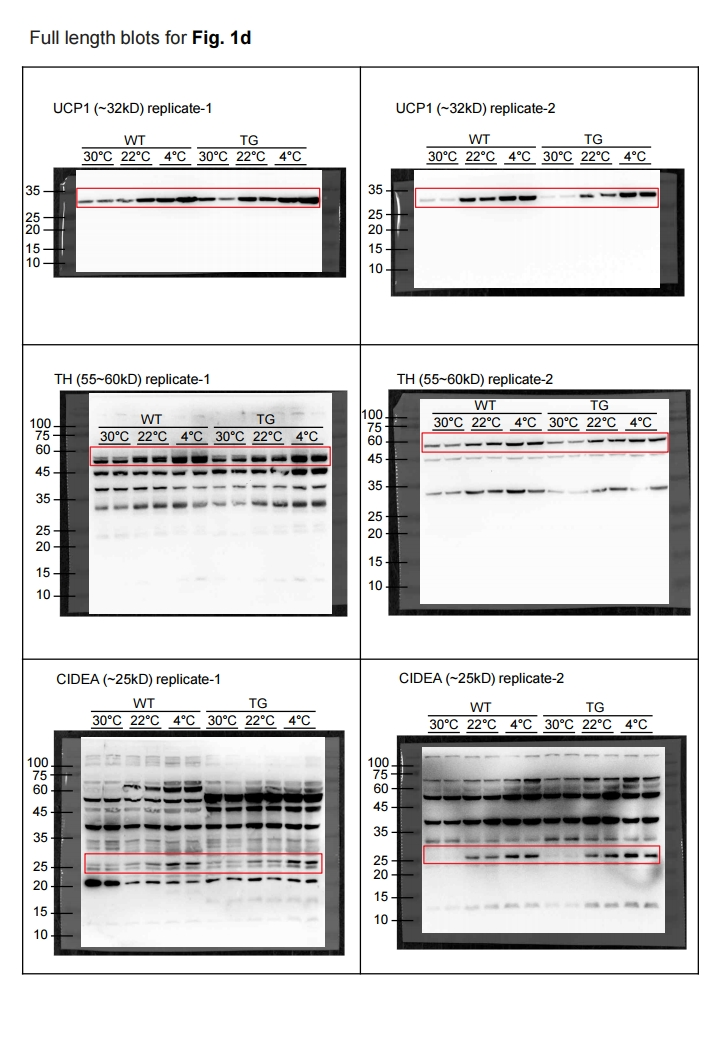

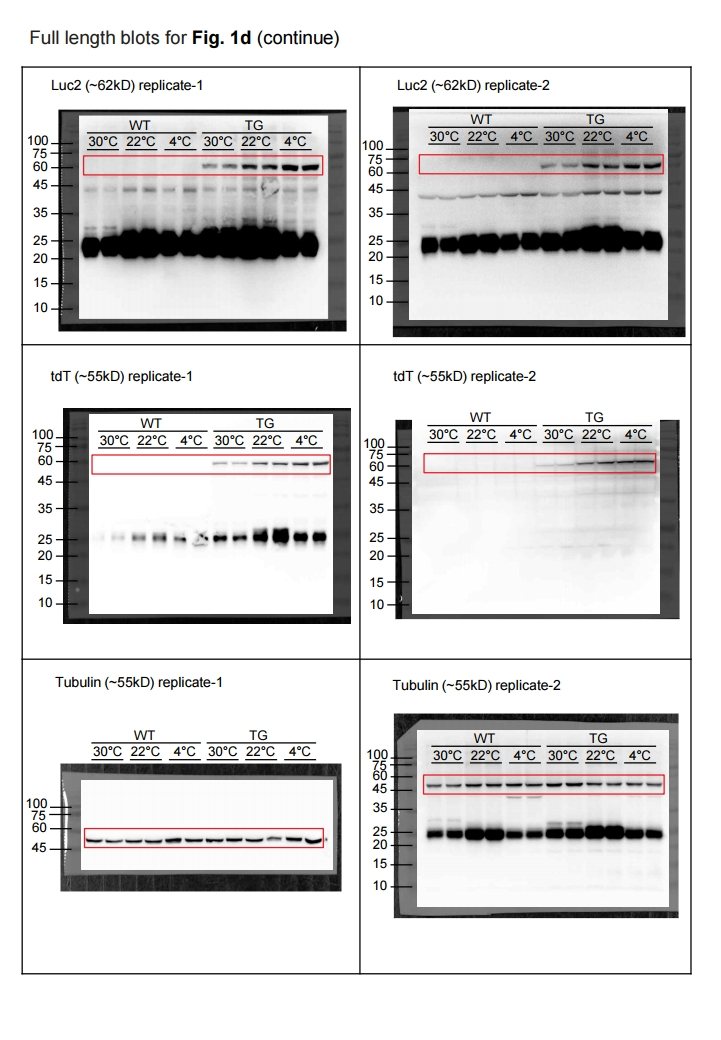

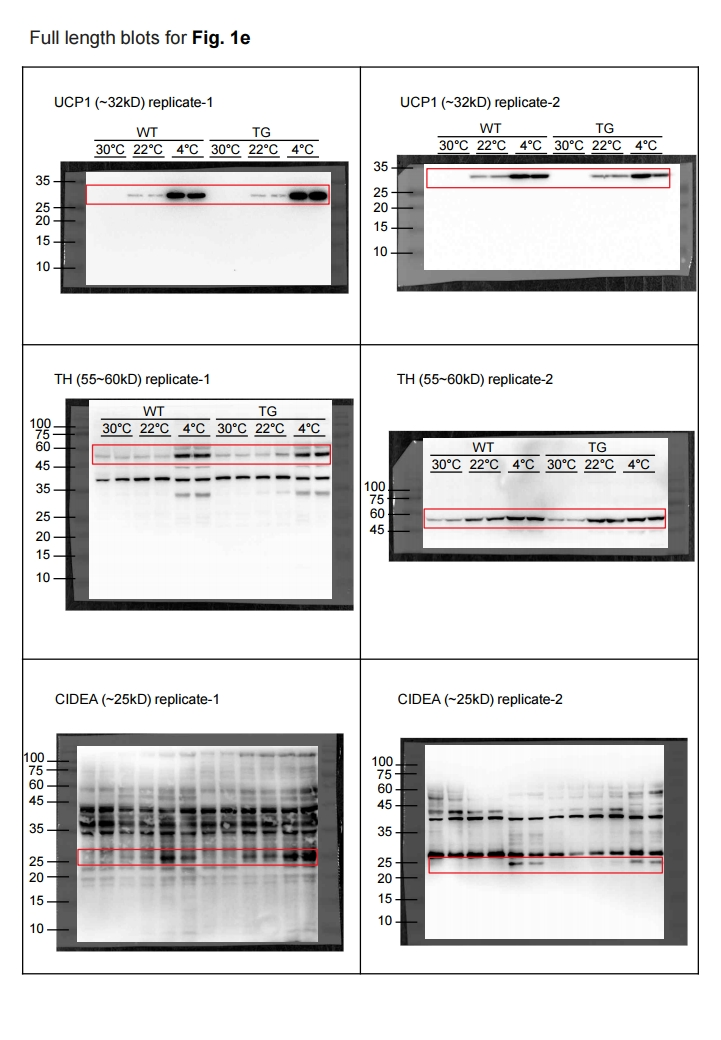

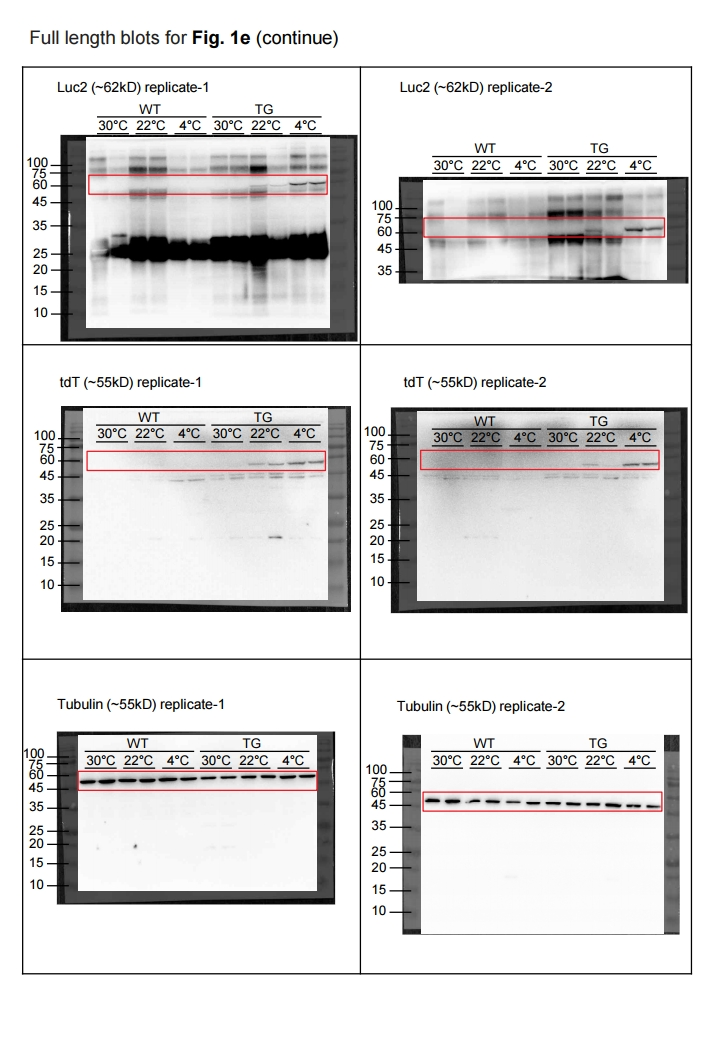

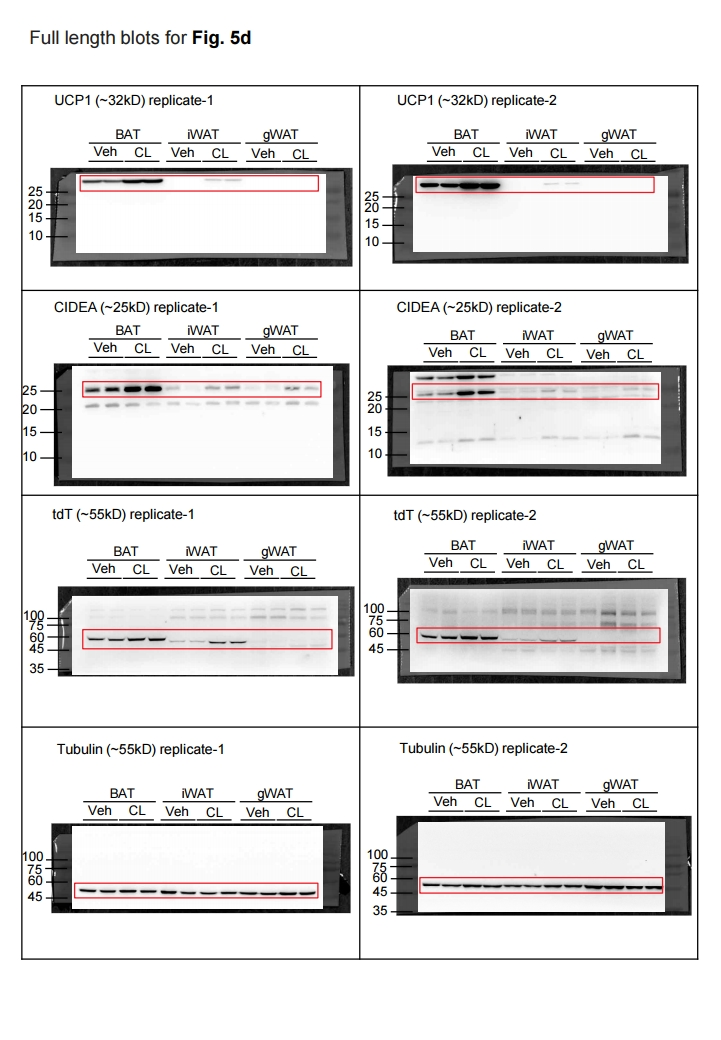

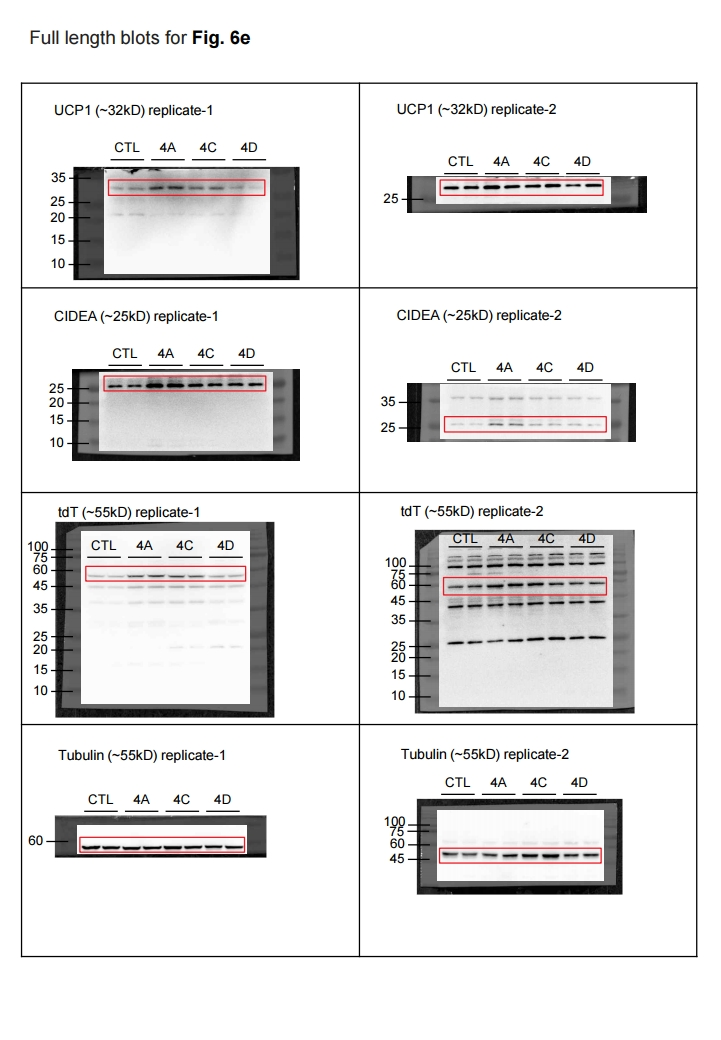

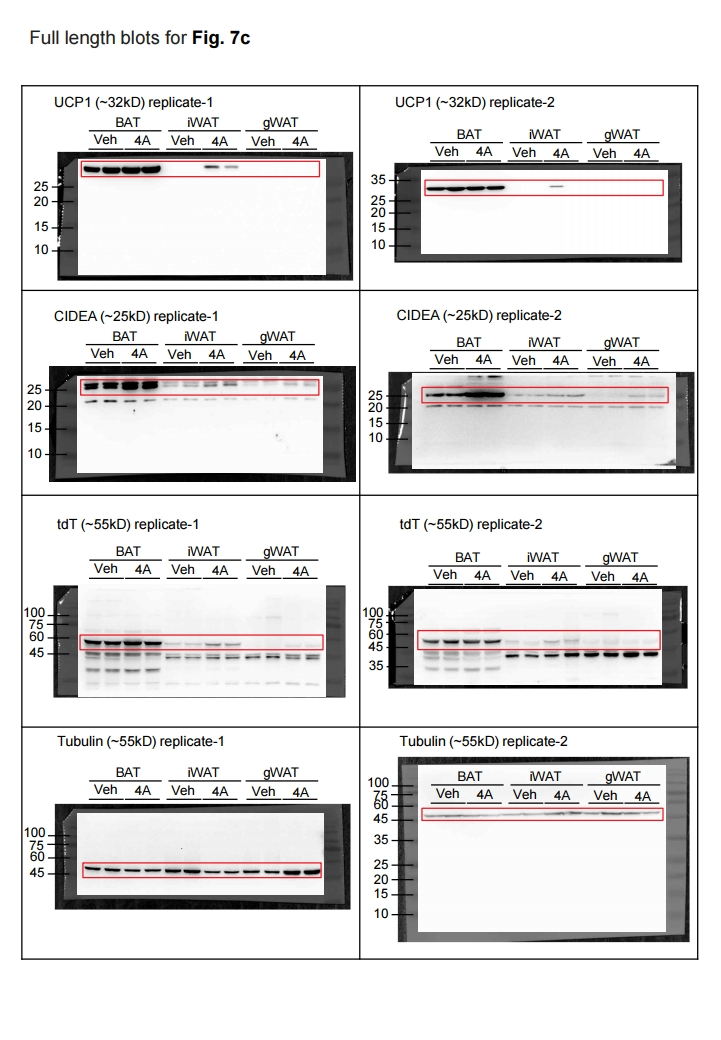

Supplement: Supplementary file 1 — Supplementary Information 1. [file 41598_2021_97959_MOESM1_ESM.docx]
